# Toll-Like Receptor 4–Myeloid Differentiation Primary Response Gene 88 Pathway Is Involved in the Inflammatory Development of Polymyositis by Mediating Interferon-γ and Interleukin-17A in Humans and Experimental Autoimmune Myositis Mouse Model

**DOI:** 10.3389/fneur.2017.00132

**Published:** 2017-04-12

**Authors:** Hongya Zhang, Fangyuan He, Ming Shi, Wenxiu Wang, Xiaojia Tian, Juan Kang, Wenjuan Han, Rui Wu, Linfu Zhou, Mengmeng Hu, Xiaobo Li, Fang Mi, Gang Zhao, Hongge Jia

**Affiliations:** ^1^Department of Neurology, Xijing Hospital, The Fourth Military Medical University, Xi’an, China; ^2^Department of Neurology, Xi’an Children’s Hospital, Xi’an, China; ^3^Department of Neurology, Shaanxi Provincial People’s Hospital, Xi’an, China; ^4^Department of Neurology, Shenzhen Hospital of Southern Medical University, Shenzhen, China

**Keywords:** polymyositis, toll-like receptor 4–MyD88 pathway, interferon-γ, interleukin-17A, EAM model

## Abstract

**Objective:**

Toll-like receptor 4 (TLR4) is one of the key players in the development of many autoimmune diseases. To determine the possible role of TLR4 in polymyositis (PM) development, we collected muscle samples from PM patients and mice subjected to an experimental autoimmune myositis (EAM) model.

**Methods:**

We measured TLR4–MyD88 pathway-related factors, interferon-γ (IFN-γ), and interleukin-17A (IL-17A) in EAM mice and PM patients. Then, we observed the changes of above factors and the inflammatory development of EAM mice with TLR4 antagonist TAK-242, IFN-γ, or IL-17A antibody treatment.

**Results:**

The expression of TLR4, MyD88, and NF-κB was significantly upregulated in the muscle tissues both in 22 patients with PM and in the EAM model. As expected, increased levels of various cytokines, such as IL-1β, IL-6, IL-10, IL-12, tumor necrosis factor-α, TGF-β, IFN-γ, and IL-17A, were evident in the serum of EAM mice. Moreover, mRNA expression levels of IFN-γ and IL-17A were significantly increased in both PM patients and EAM mice. Consistently, the levels of these factors were positively correlated with the degree of muscle inflammation in EAM mice. However, when EAM mice were treated with TLR4 antagonist TAK-242, the expression of IFN-γ and IL-17A was decreased. When the cytokines were neutralized by anti-IFN-γ or anti-IL-17A antibody, the inflammatory development of EAM exacerbated or mitigated.

**Conclusion:**

The present study provided the important evidence that the TLR4–MyD88 pathway may be involved in the immune mechanisms of PM by mediating IFN-γ and IL-17A.

## Introduction

Polymyositis (PM) is a major clinical subtype of the idiopathic inflammatory myopathies, which is classified as an autoimmune disease (1–4) and still lacks effective therapy. Females are affected more often than males at a ratio of approximately 2:1. The average age of onset of adult PM is between 52 and 56 years of age (5). The clinical symptoms are characterized by acute or subacute progressive muscle weakness, endomysia inflammatory cell infiltration, creatine kinase elevation, and abnormal electromyogram (EMG) (6). PM is histologically characterized by the presence of endomysial inflammatory infiltrates consisting of CD8^+^ T cells invading non-necrotic muscle fibers that express major histocompatibility complex class I (MHC-I) molecules on the sarcolemma (7). Thus, CD8/MHC-I complex has become a characteristic diagnostic tool for PM. However, the mechanisms underlying PM pathological process are still largely unknown.

Toll-like receptor 4 (TLR4) is an important member of the TLR family, which was discovered in *Drosophila* as a leucine-rich repeat structure that initiated the innate immune reaction and promoted the adaptive immune response (8). TLR4 is commonly expressed on mononuclear macrophages, dendritic cells, or B cells and recognizes pathogen-associated molecular patterns on microbes (9–11). TLR4 transmits signals via at least five cytosolic adaptor molecules including myeloid differentiation primary response gene 88 (MyD88), TIR domain-containing adaptor protein, interleukin-1 (IL-1) receptor-associated kinase, tumor necrosis factor (TNF) receptor-associated factor 6, and the early-phase of nuclear factor-κB (NF-κB), which leads to the induction of proinflammatory cytokines (8, 12). Once activated, the TLR4–MyD88 pathway may lead to induction of dendritic cells and inflammatory cytokines such as interferon-γ (IFN-γ) and interleukin-17A (IL-17A) (13, 14). IFN-γ is a potent activator of macrophages, which is secreted by activated T cells, NK cells, or macrophages. IL-17 family cytokines are strong inducers of inflammatory diseases, which are secreted mainly by T helper cells (15, 16).

Previous studies have shown that TLR4 may play a critical role in various autoimmune diseases (17–22). For example, the expression of TLR4 was increased in rheumatoid arthritis, systemic lupus erythematosus, and multiple sclerosis. At present, only a few reports show an increased expression of TLR4 in a small group of PM patients (23). Our previous study also showed increased expression levels of TLR4, MyD88, and NF-κB mRNA in lymph nodes of a PM animal model (24). However, the detailed changes in molecules involved in the TLR4–MyD88 signaling pathway, sequential inflammatory factors, and IFN-γ/IL-17A-producing cells during the development of PM are not fully clarified. Therefore, in the present study, we examined the expression of molecules in the TLR4 pathway and the changes of the expression of IFN-γ and IL-17A in 22 PM patients and a PM animal model, treated the mice with TLR4 antagonist TAK-242, and attempted to provide a full view of the effects of the TLR4 signal by regulating IFN-γ and IL-17A on the development of PM pathology.

## Patients and Methods

### Subjects

#### Patients and Laboratory Assessments

Muscle biopsies from patients with PM (*n* = 22), diagnosed using the classification criteria of the European Neuromuscle Centre (ENMC), were used in our study. The samples were collected from 2009 to 2012 in Xijing Hospital, the Fourth Military Medical University, Xi’an, China. The patients included 10 men and 12 women, whose mean age was 37 years (37 ± 15 years). The duration from symptom appearance to diagnosis ranged from 0.5 to 12 months. Patient data was listed in Tables 1 and 2. Muscle biopsies from six patients with periodic paralysis, showing normal histological findings, were used as the controls. Serum levels erythrocyte sedimentation rate (ESR), C-reactive protein (CRP), lactate dehydrogenase (LDH), alanine transaminase (ALT) and aspartate transaminase (AST), antinuclear antibody (ANA), the Jo-1 antibody, RO-52 antibody, anti-Sjögren’s syndrome A antibody (SSA), anti-Sjögren’s syndrome B antibody (SSB), anti-mitochondria antibody (AMA)-M2, anti-cardiolipin antibody (ACA), anti S-M2 antibody, and anti-Scl-70 antibody (Scl-70) were detected at the Department of Clinical Biochemistry Laboratory, Xijing Hospital. The EMGs were performed in the Neural Electrophysiological Laboratory of Department of Neurology, Xijing Hospital.

**Table 1:** **Clinical laboratory characteristics of patients with PM**.

Patient	Sex	Age	Symptom duration (months)	CK (IU/l)	Lactate dehydrogenase (LDH) (IU/l)	Aspartate transaminase (AST) (IU/l)	Alanine transaminase (ALT) (IU/l)	Erythrocyte sedimentation rate (ESR) (mm/h)	C-reactive protein (CRP) (mg/l)	Using Corticosteroid (Yes/No)
1	M	57	0.5	3,882	530	182	84	8	10.2	Y
2	F	32	12	2,252	669	132	103	26	0.97	N
3	F	32	12	6,311	968	411	173	30	5.28	Y
4	F	53	3	6,832	1,887	255	214	41	18.6	N
5	M	27	1	78	182	18	34	16	1.36	N
6	F	46	9	288	273	40	18	9	7.59	Y
7	M	56	8	7,421	1,260	303	223	7	11.62	Y
8	M	22	4	6,627	1,324	199	105	6	1.74	N
9	F	19	36	864	406	166	131	22	7.67	Y
10	F	25	7	3,146	960	132	98	2	2.43	Y
11	M	51	1.5	3,258	808	204	194	5	8.74	N
12	F	30	12	1,088	407	36	32	11	4.58	N
13	F	19	5	8,971	1,181	323	251	14	13.12	N
14	M	36	1	10,734	1,583	524	278	47	20.69	N
15	F	35	0.5	10,250	1,820	662	328	29	3.52	N
16	M	31	3	5,027	1,265	25	299	4	1.15	Y
17	M	36	9	1,151	295	69	65	17	21.17	N
18	F	6	1	5,107	1,467	320	266	33	6.14	N
19	F	37	5	2,288	756	182	60	36	1.42	N
20	M	40	48	15	169	29	159	12	84.14	Y
21	F	63	12	2,851	1,015	133	86	37	12.86	N
22	M	51	15	1,096	534	203	111	12	0.66	N
*N* = 22	F/M (12/10)	37 ± 15	9.3 ± 11.6	4,070.0 ± 3,322.1	898.1 ± 525.6	206.7 ± 166.7	150.5 ± 93.4	19.3 ± 13.4	11.2 ± 17.5	Y/N (8/14)

**Table 2:** **Clinical laboratory characteristics of patients with PM**.

Patient	Antinuclear antibody (ANA) (+/−)	Jo-1 (+/−)	RO-52 (+/−)	Sjögren’s syndrome A antibody (SSA) (+/−)	Sjögren’s syndrome B antibody (SSB) (+/−)	Anti-mitochondria antibody (AMA)-M2 (+/−)	Anti-cardiolipin antibody (ACA) (+/−)	S-M2 (+/−)	SCL-70 (+/−)
1	−	−	−	−	−	−	−	−	−
2	−	−	−	−	−	−	−	−	−
3	+	−	+	+	−	−	−	−	−
4	+	−	+	−	−	−	−	−	−
5	−	−	−	−	−	−	−	−	−
6	−	−	+	+	−	+	+	−	−
7	+	−	+	−	−	−	−	−	−
8	+	−	−	−	−	−	−	−	+
9	+	−	+	+	−	−	−	−	−
10	+	−	−	+	−	−	−	−	−
11	+	−	−	−	−	−	−	−	−
12	+	−	−	−	−	−	−	−	−
13	+	+	+	+	−	−	−	−	−
14	+	−	−	−	−	−	−	−	−
15	+	−	−	−	−	−	−	−	−
16	+	−	−	−	−	−	−	−	−
17	−	+	−	−	−	−	−	+	+
18	+	−	−	−	−	−	−	−	−
19	+	−	−	−	−	−	−	−	−
20	−	−	−	−	−	−	−	−	−
21	+	−	+	+	+	−	−	−	−
22	+	−	−	−	−	−	−	−	−
*N* = 22	+/− (16/6)	+/− (2/19)	+/− (7/15)	+/− (6/16)	+/− (1/21)	+/− (1/21)	+/− (1/21)	+/− (1/21)	+/− (2/20)

#### Animal Model

Female BALB/c mice at the age of 5–6 weeks (14–17 g) and a cony pig (300 g) were purchased from the Animal Center of the Fourth Military Medical University, Xi’an, China. The mice and cony pig were kept in specific pathogen-free conditions in an environmentally controlled room (23 ± 2°C, 55 ± 10% humidity) on a 12 h-light/dark cycle, fed by sterilized fodder and water *ad libitum*. During feeding and study, the body weight and health status of mice were monitored in specific pathogen-free conditions every day.

### Experimental Autoimmune Myositis (EAM) Model

Myosin was purified from the skeletal muscle of the cony pig according to Perry’s method (25) with some modifications. The EAM model was performed based on a previous report (26) with several modifications. In brief, the mice were immunized twice with a 1-week interval. Myosin (1.5 mg) and inactive *Mycobacterium tuberculosis* (5 mg/ml, BD DIFCO, 231141) in complete Freund’s adjuvant (CFA, Sigma, F5881) were injected into the muscle tissue of one limb (the first immunization) and the tail base (the second immunization). Pertussis toxin (Sigma, P2980) was injected intraperitoneally (500 ng in 200 µl normal saline) during each immunization. All experimental procedures were reviewed and approved by the Animal Studies Committee of the Fourth Military Medical University, Xi’an, China and the animal study was carried out with the established institutional guidelines regarding animal use and care.

One hundred and eighty BALB/c mice were randomly divided into five groups: the controls were normal mice. EAM mice in the other four groups were euthanized with excessive anesthesia at the end of 1–4 weeks after animals were given myosin for inducing EAM (*n* = 6, each group).

### Evaluation of Muscle Strength

Muscle strength was evaluated using an inverted screen test as described in the literature (27). The method was performed using a 50 cm^2^ screen with 1 cm^2^ mesh. Mice were placed in the center of the screen and the screen was rotated to the inverted horizontal position. The time at which the mouse fell off was noted. The evaluation of muscle strength was measured by one investigator blinded to the immunization protocol used.

### Sample Preparation

For PM patients, muscle biopsy samples were removed from one side of the musculus biceps brachii or quadriceps femoris according to a “semi-open” muscle biopsy technique. For the EAM model, muscle tissues were collected of the bilateral musculus biceps brachii and quadriceps femoris of each mouse. All the muscle samples were frozen immediately in isopentane with a container surrounded by liquid nitrogen and stored at −70°C until analysis. Blood samples of each mouse were collected in a 1.5 ml tube, centrifuged for 25 min at 4°C, and stored at −70°C for a multiplex Luminex assay.

### Histological Grading of Inflammatory Lesions and Immunohistochemistry

Muscle tissues of PM patients and EAM mice were serially cut to 10 µm thick slides. For H&E staining, the slides were stained with hematoxylin and eosin to reveal the histology of the muscle. EAM sections were analyzed according to the criterion described in a literature (25). Four parts of muscle tissue of one section were each scored individually, the mean score of which represents the inflammatory level of one mouse. All fields of each section were analyzed. The histological severity of inflammation in each muscle block was graded as follows (26, 28): grade 1, a lesion involving a single muscle fiber or less than 5 muscle fibers; grade 2, a lesion involving 5–30 muscle fibers; grade 3, a lesion involving a muscle fasciculus; and grade 4, diffuse, extensive lesions. When multiple lesions with the same grade were found in a single muscle block, 0.5 points were added to the grade. All slides were evaluated independently by two investigators who were blinded to the immunization protocol that had been used. The differences in evaluation between the two observers were resolved via conference microscopy.

For IHC, the slides were fixed in 4% paraformaldehyde for 20 min, and antigen-repaired in sodium citrate surrounded by 100°C water for 15 min. After endogenous peroxidase activity in tissues was blocked in 3% H_2_O_2_ for 30 min, the slides were incubated with the primary antibody: rabbit anti-mouse TLR4 (1:50, Abcam, ab13556), rabbit anti-human TLR4 (1:50, Santa Cruz, sc-10741), rabbit anti-mouse/human IFN-γ (1:100, proteintech, 15365-1-AP), and IL-17A (1:100, abcam, ab79056) overnight at 4°C. After washing in PBS three times, the slides were incubated with biotin-labeled goat anti-rabbit IgG (Vector Labs, BA-1000) for 2 h at room temperature, followed by streptavidin-biotin horseradish peroxidase (Vector Labs, SA-5004) for 2 h. The tissues were covered with 3,3′-diaminobenzidine tetrahydrochloride (Sigma, D5637) reagent solution for the final color product. The sections were observed with a Leica DRM microscope and Nikon imaging system.

### Real-time RT-PCR

mRNA transcripts of TLR4, MyD88, NF-κB, IFN-γ, and IL-17A were measured by RT-PCR. Total RNA from different samples was isolated in TRIZOL Reagent (Invitrogen Life Technologies, 15596-026) and reverse transcribed to cDNA with a PrimeScript^®^ RT Master Mix kit (TaKaRa, DRR036A) according to the manufacturer’s instructions. The quantitative RT-PCR was performed on a Bio-Rad iQ5TM Optical System Software Version 2.0 (Bio-Rad Laboratory) with an SYBR^®^ Premix Ex Taq™ II Kit (TaKaRa, DRR081A). The different primer sequences were listed in Table S1 in Supplementary Material. The PCR cycling parameters were 40 cycles of pre-denaturation at 95°C for 30 s, denaturation at 95°C for 10 s, and annealing at 60°C for 20 s. Data analysis was performed using the delta–delta Ct method. The results were normalized to GAPDH expression.

### Cytokine Detection

The levels of IFN-γ, IL-1β, IL-6, IL-10, IL-12, IL-17A, TNF-α, and TGF-β in mouse serum were detected using a multiplex Luminex assay according to the manufacturer’s instructions (Milliplex MAP Mouse Cytokine/Chemokine Panel-Immunology Multiplex Assay, Milliplex MAP TGFβ1-Single Plex, Millipore Corporation, Billerica, MA, USA). The plates were analyzed on the Luminex 200™ system (MAP™ Technology, Austin, TX, USA).

### EAM Mice Injected with LPS, TAK-242, Anti-IFN-γ, and Anti-IL-17A Antibodies

Experimental autoimmune myositis mice were treated by intravenous injections daily after the first immunization with the TLR4 agonist lipopolysaccharide (LPS) (2 mg/kg, sigma, L2880) or theTLR4 antagonist TAK-242 (3 mg/kg, CALBIOCHEM, 614316) for 14 days. EAM mice were treated by intraperitoneal injections of anti-IFN-γ (1.0 mg/mouse, weekly, Biolegend, 506963) and anti-IL-17A (100 μg/mouse, every 3 days, Biolegend, 505827) antibodies after the first immunization for 14 days. The mice were sacrificed 7 days after the last immunization. MyD88, NF-κB, IFN-γ, and IL-17A mRNA in muscle tissue were measured by RT-PCR. IFN-γ and IL-17A levels in serum were detected by Luminex.

### Statistics

All the data were shown as the mean ± SD, and differences between two groups were analyzed by one-way classification ANOVA and multiple groups by LSD using SPSS19.0 software. Normal distribution variance data between two groups were analyzed using a Pearson linear correlation, and a non-normal distribution variance using Spearman rank correlation. Significance was considered at *P* < 0.05.

## Results

### Clinical and Laboratory Features of PM Patients

Our data from PM patients showed elevated levels of serum ESR, CRP, LDH, ALT, and AST, some of which were over 10 times the normal ceiling value (Table 1). Although PM is often diagnosed by clinical symptoms, kinase examination, and muscle biopsy, more and more studies indicated that autoantibodies also existed in the PM patients (5, 29, 30). Consistently, of the 22 PM patients in our study, 16 patients expressed ANA, 2 expressed the Jo-1 antibody, 7 expressed RO-52 antibody, 6 expressed SSA antibody, 1 expressed SSB antibody, 1 expressed AMA-M2, 1 expressed ACA, 1 expressed anti S-M2 antibody, and 2 expressed Scl-70 antibody (Table 2).

The EMGs of all PM patients revealed abnormal findings: increased insertional and spontaneous activity in the form of fibrillation potentials, positive sharp waves, complex repetitive discharges, short duration, small amplitude, or polyphasic motor unit action potentials (data not shown).

### Changes in Expression of TLR4, MyD88, or NF-κB in PM Muscle Tissues

**Figure 1:**
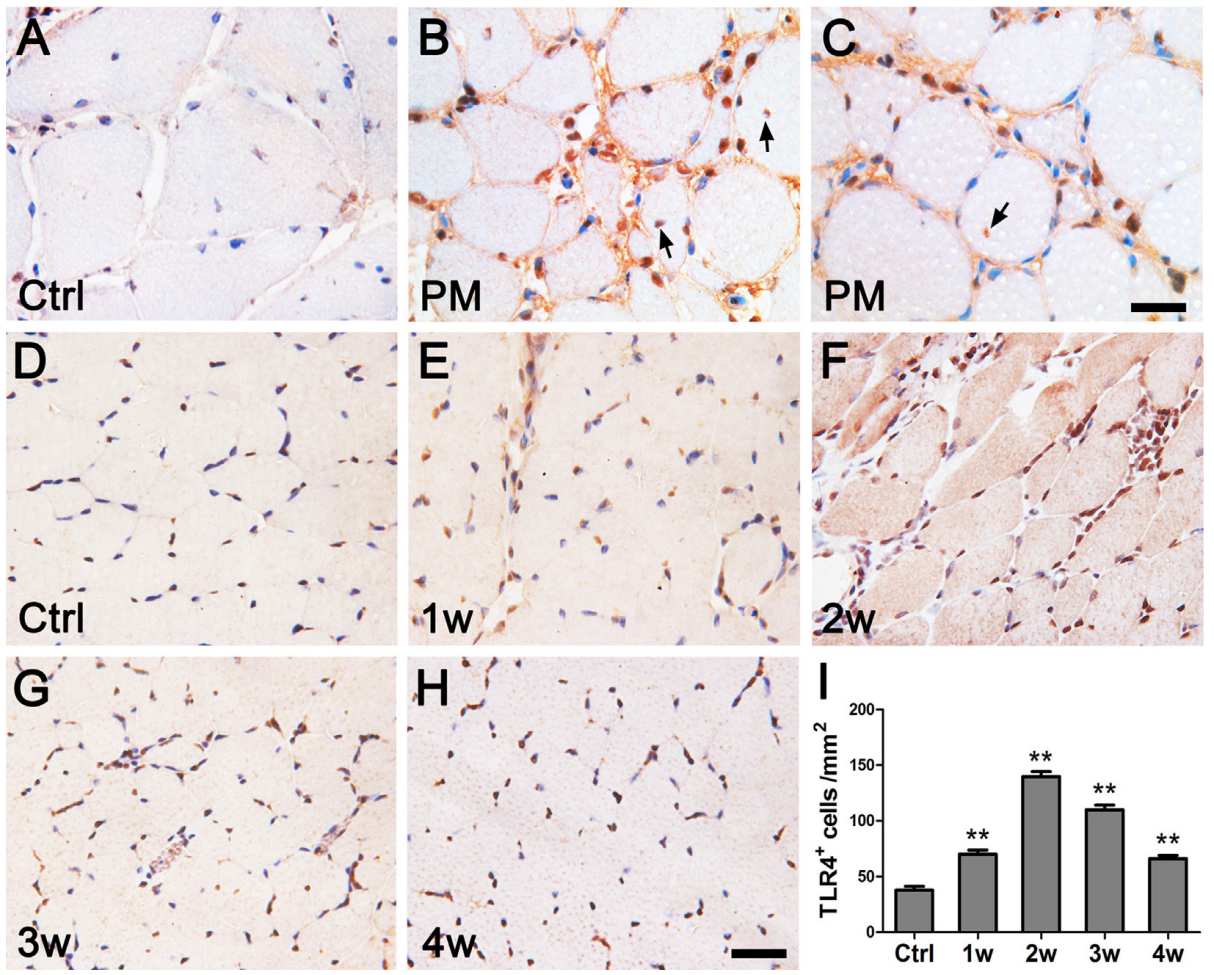
**Immunohistochemistry of toll-like receptor 4 (TLR4) in polymyositis (PM) patients and experimental autoimmune myositis (EAM) mice**. **(A–C)** In muscle tissues of patients with PM, compared with the controls [Ctrl (A)], TLR4 was strongly expressed by infiltrating cells surrounding the muscle bundle, on the endomysial, and in the muscle fibers [indicated by arrows in **(B,C)**]. **(D–I)** In muscle tissues of mice with EAM, TLR4 expression was weak in the controls **(D)**, began to be upregulated at 1 week after the first immunization **(E)**, reached to the peak at 2 weeks **(F)**, and attenuated at 3 weeks **(G)** and 4 weeks **(H)**. The nuclei were counterstained with hematoxylin. **P < 0.001 vs. the controls (Ctrl). Scale bars: 50 µm.

IHC showed that TLR4^+^ cells were present in the muscle fibers, endomysial, and around muscle bundles in the muscle tissues of PM patients, whereas no obvious TLR4^+^ cells were observed in the controls (Figures 1A–C). TLR4 expression was elevated in EAM mice compared to the controls, and the maximal level was observed after the first immunization (Figures 1D–I).

**Figure 2:**
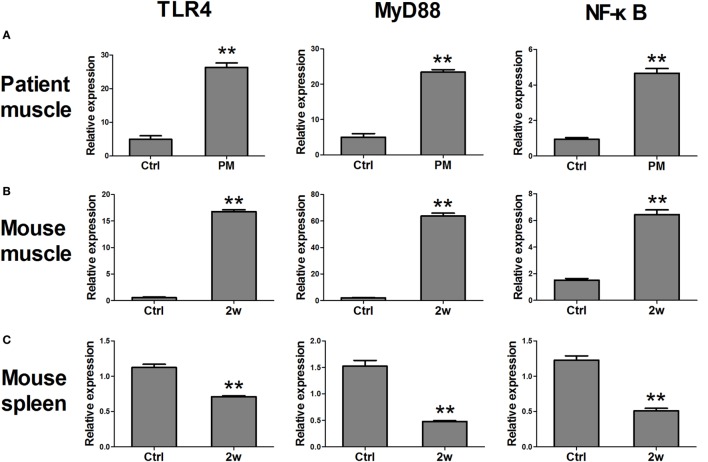
**The changes in mRNA expression of toll-like receptor 4 (TLR4) pathway-related molecules in polymyositis (PM) patients and experimental autoimmune myositis (EAM) mice**. **(A)** mRNA expression of TLR4, MyD88, and NF-κB were significantly increased in muscle tissues of biopsy samples from PM patients compared with the controls. **(B)** Compared with the controls, mRNA expression of TLR4, MyD88, and NF-κB were elevated in muscle tissues of EAM mice at 2 weeks after the first immunization. **(C)** Compared with the controls, mRNA expression of TLR4, MyD88, and NF-κB were decreased in the spleen of EAM mice at 2 weeks. **P < 0.001 vs. the controls (Ctrl).

Real-time PCR assays were performed to confirm the results of the IHC. Our results showed that the relative mRNA levels of TLR4 (26.28 ± 6.41, *P* < 0.001) and its downstream factors MyD88 (23.43 ± 3.33, *P* < 0.001) and NF-κB (4.67 ± 1.24, *P* < 0.001) were significantly upregulated in PM patients compared to those of their respective controls (4.97 ± 2.59, 4.99 ± 2.50, and 0.94 ± 0.23) (Figure 2A). Similar results were also observed in EAM mice. At 2 weeks after the first immunization, the relative mRNA levels of TLR4 (16.77 ± 0.93, *P* < 0.001), MyD88 (63.71 ± 5.30, *P* < 0.001), and NF-κB (6.45 ± 0.86, *P* < 0.001) were significantly elevated compared with their respective controls (0.54 ± 0.26, 2.00 ± 0.58, and 1.53 ± 0.29) (Figure 2B). It was noted that at 3 or 4 weeks after the first immunization, the expression of TLR4, MyD88, and NF-κB was recovered but still higher than that of the controls. Interestingly, in contrast to muscle tissue, TLR4 transcripts (0.71 ± 0.04, *P* < 0.001), MyD88 (0.48 ± 0.04, *P* < 0.001), and NF-κB (0.51 ± 0.09, *P* < 0.001) were significantly decreased in the spleen of EAM mice compared with controls (1.12 ± 0.11, 1.52 ± 0.28, 1.23 ± 0.15) (Figure 2C). Therefore, these results showed increased levels of TLR4, MyD88, and NF-κB in the muscle tissues of PM patients and EAM mice.

### Changes in Expression Levels of Cytokines in EAM Mice

**Figure 3:**
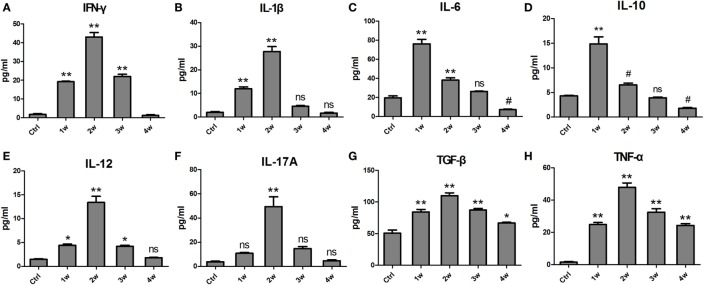
**The changes in levels of inflammatory cytokines in the serum of experimental autoimmune myositis (EAM) mice at 1–4 weeks after the first immunization**. The concentrations of IFN-γ **(A)**, IL-1β **(B)**, IL-6 **(C)**, IL-10 **(D)**, IL-12 **(E)**, IL-17A **(F)**, TGF-β **(G)**, and tumor necrosis factor (TNF)-α **(H)** were detected by a Luminex assay. **P < 0.001, *P < 0.01, ^#^*P* < 0.05, ^ns^*P* > 0.05, vs. the controls (Ctrl).

Liquid chip assays were used to examine the levels of various cytokines in the serum of EAM mice at 1 ~ 4 weeks after the first immunization. At 1 week, the levels of IL-6 (76.13 ± 11.91 pg/ml), and IL-10 (14.87 ± 3.47 pg/ml) were prominently elevated compared with controls (19.68 ± 5.63, 4.34 ± 0.30 pg/ml, *P* < 0.001). At 2 weeks, IFN-γ (43.05 ± 5.97 pg/ml), IL-1β (27.7 ± 5.25 pg/ml), IL-12 (13.39 ± 3.11 pg/ml), IL-17 A (49.35 ± 19.87 pg/ml), TNF-α (47.87 ± 6.70 pg/ml), and TGF-β (110.00 ± 12.17 ng/ml) reached their maximal levels compared with the controls (1.66 ± 0.98, 1.98 ± 0.90, 1.52 ± 0.24, 3.86 ± 0.70, 1.68 ± 1.03, 50.50 ± 13.55, *P* < 0.001). At 3 weeks, the cytokine levels decreased but were still higher than those of the controls. Most of the cytokines except TNF-α almost recovered to control levels at 4 weeks (Figure 3). Overall, the levels of cytokines were elevated 1–3 weeks after the first immunization.

### Involvement of IFN-γ and IL-17A in PM Patients and EAM Mice

**Figure 4:**
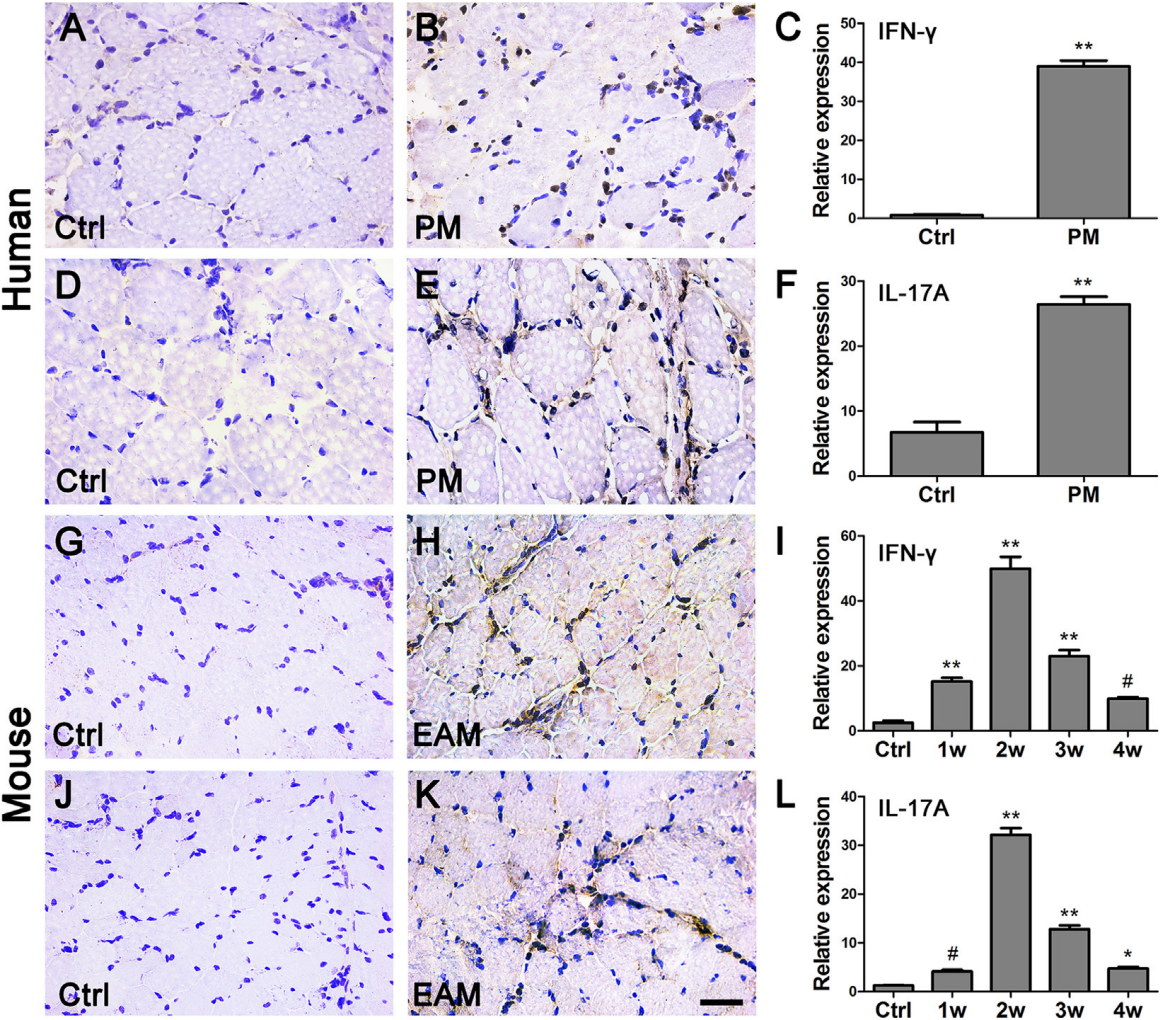
**The changes of interferon-γ (IFN-γ) and interleukin-17A (IL-17A) in polymyositis (PM) patients and experimental autoimmune myositis (EAM) mice**. **(A–F)** As compared with the controls (Ctrl), the expression of IFN-γ and IL-17A in the biopsy samples from PM patients were increased. **(G–L)** Compared with the controls, the expression of IFN-γ and IL-17A in the muscle of EAM mice were significantly increased. **P < 0.001, *P < 0.01, ^#^*P* < 0.05, vs. the controls (Ctrl). The nuclei were counterstained with hematoxylin. Scale bars: 50 µm.

Various inflammatory cytokines could promote the differentiation from naïve T cells into functional T cells, which as well as macrophages are involved in various autoimmune diseases by secreting IFN-γ and IL-17A and so on (16). The mRNA levels of IFN-γ (39.02 ± 6.86) and IL-17A (26.43 ± 5.49) were elevated, respectively, in the muscle tissue of PM patients compared with the controls (0.87 ± 0.35, 6.74 ± 3.87, respectively, *P* < 0.001) (Figures 4C,F). Similar results were also found in the EAM mice. At 2 weeks after the first immunization, the relative mRNA levels of IFN-γ (49.98 ± 8.83) and IL-17A (32.15 ± 3.44) were markedly increased compared with their respective controls (2.54 ± 1.50 and 1.25 ± 0.21, respectively, *P* < 0.001) (Figures 4I,L). At 3 or 4 weeks after the first immunization, their expression was attenuated but still higher than that of the controls. Moreover, IHC showed that IFN-γ^+^ and IL-17A^+^ cells are increased in the PM patients (Figures 4B,E) and EAM mice at 2 weeks(Figures 4H,K) compared with the controls (Figures 4A,D,G,J). Unexpectedly, the expression of these factors in the spleen was decreased compared with their respective controls (Figure S1 in Supplementary Material). Therefore, our results revealed that IFN-γ and IL-17A producing cells may be involved in the development of PM.

### Correlations between PM Severity and TLR4-Related Factors, IFN-γ, or IL-17A

**Figure 5:**
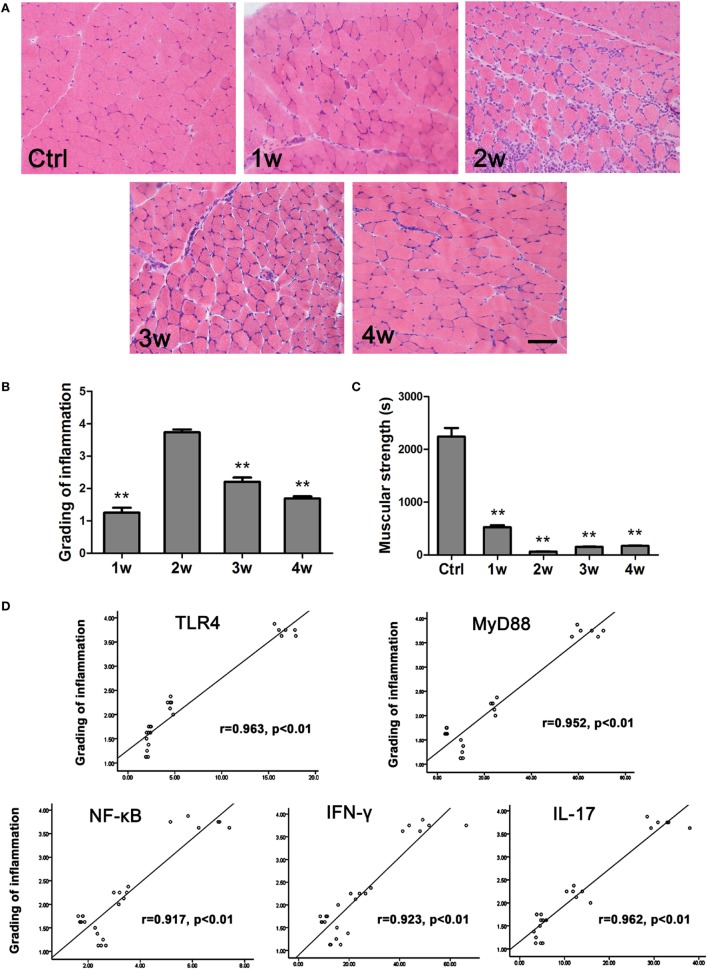
**The correlations of PM severity with toll-like receptor 4 (TLR4)–MyD88 pathway, interferon-γ (IFN-γ), and interleukin-17A (IL-17A)**. **(A)** HE stain showed the infiltrating cells in the muscle of experimental autoimmune myositis (EAM) mice at 1–4 weeks after the first immunization. Scale bar: 100 µm. **(B)** Muscle grading of inflammation at different time points (Ctrl). **(C)** Evaluation of muscular strength. **P < 0.001, vs. the controls. **(D)** The expression of TLR4, MyD88, NF-κB, IFN-γ, or IL-17A had a positive correlation with muscle grading of inflammation in EAM mice, respectively.

To determine the severity of EAM in the mice, inflammatory lesion grading was evaluated according to a previous report (see the Methods section). HE staining results showed that at 2 weeks after the first immunization, more inflammatory cells and damaged muscle fibers were evident than at 1, 3, or 4 weeks (Figure 5A). Histological grading of inflammatory lesions was shown in Figure 5B. Consistent with these results, at 2 weeks, mouse muscle strength indicated by the duration a mouse staying in the inverted screen (63.13 ± 6.42 s) was worse than that of the controls (2,241.8 ± 162.63 s, *P* < 0.001) and other groups (1 week, 527.15 ± 33.86 s, *P* < 0.001; 3 weeks, 152.98 ± 3.45 s, *P* = 0.047; 4 weeks, 171.97 ± 3.36 s, *P* = 0.018) (Figure 5C). We performed a Pearson linear correlation to analyze the correlations of PM severity with TLR4-related factors, including TLR4, MyD88, NF-κB, IFN-γ, or IL-17A expression, respectively. Our results showed that the mRNA expression levels of TLR4, MyD88, NF-κB, IFN-γ, or IL-17A in muscle tissue of EAM mice were positively correlated with grading inflammatory lesions (*r* = 0.963, *r* = 0.952, *r* = 0.917, *r* = 0.923, *r* = 0.962, respectively, *P* < 0.001) (Figure 5D).

### Requirement of TLR4–MyD88 Signaling in the Development of PM

**Figure 6:**
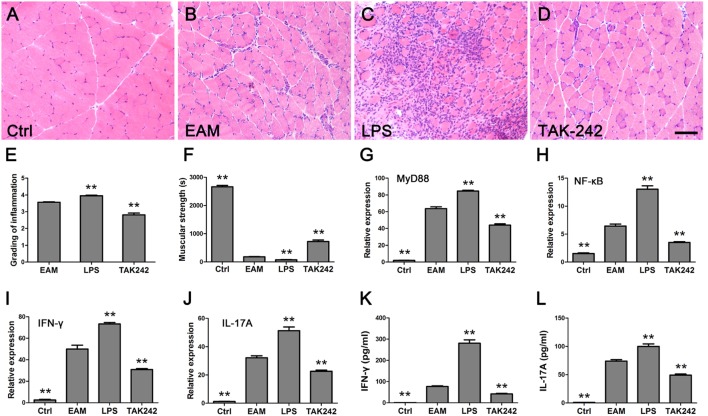
**Toll-like receptor 4 (TLR4)–MyD88 pathway was required in polymyositis (PM)**. **(A–D)** HE stain showed the infiltrating cells in the muscle of normal mice and experimental autoimmune myositis (EAM) mice treated with normal saline only, lipopolysaccharide (LPS) or TAK-242, respectively. Scale bar: 100 µm. The grading evaluation of inflammation was decreased and the muscular strength was stronger in EAM mice treated with TAK-242 **(E,F)**. The mRNA expression of MyD88, NF-κB, IFN-γ, and IL-17A in muscle tissues of mice were upregulated after treatment with LPS and downregulated after treatment with TAK-242 [**(G–J)**, respectively]. IFN-γ and IL-17A in the serum of mice increased or decreased after treatment with LPS or TAK-242, respectively **(K,L)**. **P < 0.001, versus the EAM mice treated with normal saline only.

Part of TLR4 signaling is dependent on MyD88 (8). In muscle tissue of EAM mice, the TLR4 agonist LPS exacerbated the development of PM (Figures 6A–C), and upregulated the expression of MyD88 (84.60 ± 2.61), NF-κB (13.01 ± 1.51), IFN-γ (73.30 ± 3.46), and IL-17A (51.28 ± 6.48), compared with the EAM group treated with normal saline only (63.71 ± 5.30, 6.45 ± 0.86, 49.98 ± 8.83, 32.15 ± 3.44, respectively, *P* < 0.001) (Figures 6G–J). However, the TLR4 antagonist TAK-242 mitigated the inflammatory development of PM (Figures 6A,B,D), and downregulated the expression of MyD88 (44.02 ± 3.70), NF-κB (3.51 ± 0.37), IFN-γ (30.87 ± 2.38), and IL-17A (22.60 ± 2.01), compared with the EAM group treated with normal saline only (63.71 ± 5.30, 6.45 ± 0.86, 49.98 ± 8.83, 32.15 ± 3.44, respectively, *P* < 0.001) (Figures 6G–J). Consistently, the levels of IFN-γ and IL-17A were increased or decreased in the serum of EAM mice treated with LPS (281.14 ± 38.78, *P* < 0.001; 99.94 ± 11.06, *P* < 0.001) or TAK-242 (41.99 ± 6.47, *P* = 0.007; 49.20 ± 4.52, *P* < 0.001), respectively, compared with the normal saline group (76.78 ± 7.14, 74.07 ± 5.91) (Figures 6K,L). Together, these data indicated that TLR4 signaling was involved in the PM and played a pivotal role in EAM.

### Involvement of TLR4-Mediated IFN-γ and IL-17A in the Development of EAM

**Figure 7:**
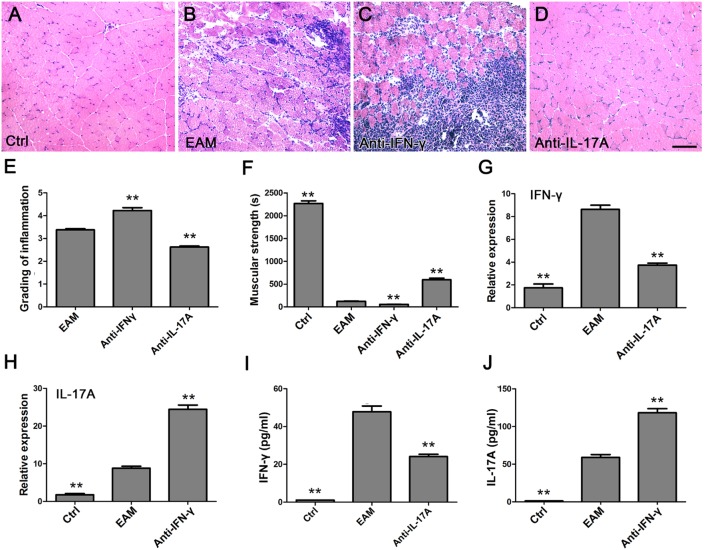
**Different roles of toll-like receptor (TLR4)-mediated interferon-γ (IFN-γ) and interleukin-17A (IL-17A) in the course of development of experimental autoimmune myositis (EAM)**. **(A–D)** HE stain showed the infiltrating cells in the muscle of normal mice and EAM mice treated with normal saline only, anti-IFN-γ, or anti-IL-17 antibody, respectively. Scale bar: 100 µm. The grading evaluation of inflammation was increased or decreased but the muscular strength was weaker or stronger in EAM mice-treated anti-IFN-γ or anti-IL-17 antibody, respectively **(E,F)**. The mRNA expression of IFN-γ was decreased but IL-17A increased in muscle tissues of EAM mice-treated anti-IL-17 or anti-IFN-γ antibody, respectively **(G,H)**. IFN-γ or IL-17A was decreased or increased in the serum of mice-treated anti-IL-17 or anti-IFN-γ antibody, respectively **(I,J)**. **P < 0.001, vs. the EAM mice treated with normal saline only.

The expression of TLR4-mediated IFN-γ and IL-17A was elevated in muscle tissue of PM patients and EAM mice as well as in the EAM serum, implying their important roles in the PM. To confirm this point, EAM mice were injected intraperitoneally with anti-IFN-γ antibody or anti-IL-17A antibody. The grading of inflammation increased in EAM mice treated with IFN-γ antibody (4.23 ± 0.30) (Figures 7C,E) but decreased with IL-17A antibody (2.63 ± 0.11) (Figures 7D,E) significantly compared with EAM mice (3.38 ± 0.14, *P* < 0.001) (Figures 7B,E). However, the EAM mice treated with anti-IFN-γ antibody had weaker muscular strength (60.85 ± 6.27) but had stronger muscular strength (595.00 ± 85.73) with anti-IL-17A antibody treatment compared with EAM mice (121.07 ± 18.52, *P* < 0.001) (Figure 7F). The expression of IFN-γ mRNA (3.75 ± 0.41) in the muscle tissue and IFN-γ (24.13 ± 2.93 pg/ml) in the serum were downregulated with IL-17A antibody compared with EAM mice (8.63 ± 0.90, 47.89 ± 7.32, respectively, *P* < 0.001) (Figures 7G,I) but IL-17A mRNA(24.47 ± 2.72) and IL-17A(118.33 ± 13.01 pg/ml) treated with anti-IFN-γ antibody were upregulated compared with EAM mice (8.82 ± 1.28, 59.08 ± 9.18, respectively, *P* < 0.001) (Figures 7H,J). These data suggested that TLR4-mediated IFN-γ and IL-17A may be responsible for the inflammatory development of EAM.

## Discussion

A group of studies have demonstrated that TLR4 plays an important role in autoimmune diseases (15). In this work, we showed a possible involvement of TLR4 and its downstream effectors, MyD88 and NF-κB, in the development of PM in human patients and in an EAM mouse model. Furthermore, we found that IFN-γ- and IL-17A-producing cells were involved and dependent on the TLR4–MyD88 pathway in the pathological progression of PM.

Toll-like receptor 4 was proposed to play an important role in various autoimmune diseases, such as rheumatoid arthritis, systemic lupus erythematosus, and multiple sclerosis (19–21). Our previous study also showed increased expression levels of TLR4 and its downstream effectors in the lymph nodes in a PM animal model (24). As an extended study on PM pathology, here, we analyzed the changes in TLR4 expression using muscle biopsies from 22 PM patients and EAM mice. In PM patients, TLR4 expression was significantly increased compared with that of patients with periodic paralysis. Our results were consistent with the previous reports (23, 31). In EAM mice, we revealed a dynamic change of TLR4 expression, which reached a peak 2 weeks after the first immunization. Furthermore, we analyzed changes in the expression of MyD88 and NF-κB, two key downstream effectors of TLR4. Similarly, in both PM patients and EAM mice, the expression of MyD88 and NF-κB was significantly increased as well (Figures 1 and 2).

It is known that the TLR4–MyD88 pathway involves the production of various inflammatory cytokines. Therefore, we also investigated the changes of cytokine expression in the serum of EAM mice. As expected, our results showed that the levels of IFN-γ, IL-1β, IL-6, IL-10, IL-12, IL-17A, TNF-α, and TGF-β were markedly elevated (Figure 3). However, a previous study showed the reduced Th1 and Th17 cells in PMBCs from PM patients (32). We proposed that this discrepancy might attribute to the differences between PM patients and PM mouse model. In animal models, increased IFN-γ-producing cells and IL-17A-producing cells were also observed in previous studies (33–37). Additionally, it was noted that IL-6 and IL-10 reached their maximal at 1 week after the first immunization whereas others reached their maximal level at 2 weeks. We proposed that IL-6 and IL-10 may play an important role in the early stages of PM progression (38).

Various cytokines can induce the differentiation of naïve T cells into different subpopulations (16, 39). It was reported that IL-6 and TGF-β together may drive the differentiation of naïve T cells into Th17 cells (14, 40), and IL-6 and IFN-γ may be involved in Th1 differentiation (34, 41). In the present study, the expression of IFN-γ and IL-17A in muscle tissues of EAM mice was elevated (Figure 4) at a time that coincided with elevated expression of TLR4, MyD88, NF-κB as well as various cytokines (except IL-6 and IL-10). Moreover, we found that the expression of TLR4, MyD88, NF-κB, IFN-γ, and IL-17A was positively correlated with the grading of muscle inflammation in EAM mice (Figure 5D). Therefore, our results suggested that the TLR4–MyD88 pathway and IFN-γ and IL-17Acells may play an important role in the development of PM.

Interestingly, our study showed that in the spleen of EAM mice, the expression of TLR4–MyD88 related molecules, IFN-γ, and IL-17A (Figure 2; Figure S1 in Supplementary Material) was decreased compared with the controls. Moreover, the expression was lowest at 2 weeks after immunization. These findings were converse to those in muscle tissue and have not been reported elsewhere. We presumed that these molecules are relocated from spleen to muscle by blood circulation or lymph recirculation, which could distribute lymphocytes between lymphoid tissue and other organs, arouse lymphocytes to remove invading pathogens, and promote antigen-activated lymphocytes to drain into local lymphoid tissue or organs. There, T cells, B cells, and APC may collaborate in the immune response for the production of effective lymphocytes (38, 42). The effective lymphocytes could migrate directly and intensively to the site of inflammation to induce an immune response (43, 44).

Geun-Tae Kim et al. (23) reported that the expression of TLR4 was increased in few PM patients. In our research, muscle tissue samples of 22 people with PM were studied. It was shown that the expression levels of TLR4 protein and TLR4 mRNA in muscle tissues of PM patients were significantly increased compared with the controls (Figures 1 and 2). Additionally, the relative expression levels of IFN-γ and IL-17A in muscle tissues of patients with PM were predominantly elevated compared with controls (Figure 4). This supported our conclusions from the EAM model.

These results were consistent with the inflammatory lesion grading trends of EAM mice. Moreover, we observed that muscle inflammation of EAM mice occurred at the same time point that EAM mouse strength was weakest (Figure 5) and the expression of TLR4 mRNA was highest (Figure 2). This trend suggested that TLR4 may trigger PM in the early immune responses (Figure 5). Based on above results, we used TLR4 agonist LPS and antagonist TAK-242 and found that TLR4 signaling was required in the development of PM. Furthermore, we revealed that TLR4-mediated IFN-γ and IL-17A played an important role in the inflammatory development of EAM, by using IFN-γ and IL-17A neutralization antibodies (Figures 6 and 7). Additionally, our results suggested that IFN-γ could alleviate but IL-17A could aggravate the inflammatory response (Figure 7).

Additionally, we still lacked the knowledge about how TLR4 was activated in our EAM model. It is reported that exogenous sources (e.g., LPS) and endogenous TLR4 ligands have been identified from both the host tissues and cells. The endogenous intracellular triggers of TLR4 mainly include the DNA-binding protein high-mobility group box 1 (HMGB1) and cellular heat shock proteins (45). Previous studies have showed that HMGB1 takes part in driving the inflammation of PM patients (46, 47). Therefore, we proposed that HMGB1 may be involved in our EAM model, and admittedly further studies should be done to address this question.

Another question to be addressed in this study is whether TLR4 pathway is related to the autoantibodies present in the PM patients (Table 2). Traditionally, PM is proposed to be stem from cellular immunity not humoral immunity. Previous reports (5, 29, 30) and our present studies revealed the presence of autoantibodies in the PM patients. We still lack knowledge about the correlation between TLR4 pathway and autoantibodies. However, we propose that the TLR4 pathway may induce T helper cell-expressing cytokines to act on B cells indirectly, and subsequently promote the production of autoantibodies. Additionally, genetic aspects (e.g., HLA) were associated with PM development (48–52) which may reflect the autoantibody profiles. In the present study, however, all the patients had no obvious genetic history in the family. Association of genetic factors with PM will be the direction of our future work.

In this work, by using the samples from PM patients and the EAM model, we found that TLR4 signaling may play a crucial role in triggering the inflammation of PM, and this process required the involvement of TLR4-mediated IFN-γ and IL-17A (Figure S2 in Supplementary Material). To some extent, this work partly documented the immune mechanism of PM and supplied a new direction for the development of PM therapy.

## Ethics Statement

This study was approved by Institutional Board of the Fourth Military Medical University, Xi’an, China and conducted in accordance with Declaration of Helsinki. All muscle biopsies were obtained according to current ethics regulations and the written informed consent was obtained from all subjects. The proposed protocol conforms to the Good Clinical Practice.

## Author Contributions

All the authors designed the study, performed the study, wrote the manuscript, and read and approved the final version of the paper.

## Conflict of Interest Statement

The authors declare that the research was conducted in the absence of any commercial or financial relationships that could be construed as a potential conflict of interest.

## Abbreviations

PM, polymyositis; EAM, experimental autoimmune myositis; TLR4, toll-like receptor 4; MyD88, myeloid differentiation primary response gene 88; NF-κB, nuclear factor-κB; IFN-γ, interferon-γ; IL-17A, interleukin-17A.
