# Supplementary material for: Toll-Like Receptor 4–Myeloid Differentiation Primary Response Gene 88 Pathway Is Involved in the Inflammatory Development of Polymyositis by Mediating Interferon-γ and Interleukin-17A in Humans and Experimental Autoimmune Myositis Mouse Model
Source: Front Neurol. 2017 Apr 12;8:132. doi: 10.3389/fneur.2017.00132 (PMC5388689; doi:10.3389/fneur.2017.00132)
Supplement: Supplementary file 1 [file Table_1.DOCX]

| Gene name | Primers( mouse) | Primers (human) |
| --- | --- | --- |
| GAPDH | Forward 5´- TGTGTCCGTCGTGGATCTGA-3´ | Forward 5´- GCACCGTCAAGGCTGAGAAC-3´ |
|  | Reverse 5´-TTGCTGTTGAAGTCGCAGGAG-3´ | Reverse 5´- TGGTGAAGACGCCAGTGGA-3´ |
| TLR4 | Forward 5´-GGGCCTAAACCCAGTCTGTTTG-3´ | Forward 5´- CTGGGTGTGTTTCCATGTCTCA-3´ |
|  | Reverse 5´-CTTCTGCCCGGTAAGGTCCA-3´ | Reverse 5´- TGCGGACACACACACTTTCAAATA-3´ |
| MyD88 | Forward 5´-AAGATGACCCTGGGAGCCCTA-3´ | Forward 5´- GCACATGGGCACATACAGAC-3´ |
|  | Reverse 5´-CTCAGGCCAGTCATCATTGAACA-3´ | Reverse 5´- GACATGGTTAGGCTCCCTCA-3´ |
| NF-κB | Forward 5´- ACCACTGCTCAGGTCCACTGTC-3´ | Forward 5´- GCCTCCACAAGGCAGCAAATA-3´ |
|  | Reverse 5´-GCTGTCACTATCCCGGAGTTCA-3´ | Reverse 5´- CACCACTGGTCAGAGACTCGGTAA-3´ |
| IFN-γ | Forward 5´-CGGCACAGTCATTGAAAGCCTA-3´ | Forward5´-CTTTAAAGATGACCAGAGCATCCAA-3´ |
|  | Reverse5´-GTTGCTGATGGCCTGATTGTC-3´ | Reverse 5´-GGCGACAGTTCAGCCATCAC-3´ |
| IL-17A | Forward 5´-GGAAAGCTGGACCACCACA-3´ | Forward5´-TCTGTGATCTGGGAGGCAAA-3´ |
|  | Reverse 5´-CACACCCACCAGCATCTTCTC-3´ | Reverse5´-CTCTTGCTGGATGGGGACA-3´ |

**Supplementary Table 1. Sequences of oligonucleotides used in this study**
