# Supplementary material for: Toll-Like Receptor 4–Myeloid Differentiation Primary Response Gene 88 Pathway Is Involved in the Inflammatory Development of Polymyositis by Mediating Interferon-γ and Interleukin-17A in Humans and Experimental Autoimmune Myositis Mouse Model
Source: Front Neurol. 2017 Apr 12;8:132. doi: 10.3389/fneur.2017.00132 (PMC5388689; doi:10.3389/fneur.2017.00132)
Supplement: Supplementary file 2 [file Image_1.PDF]

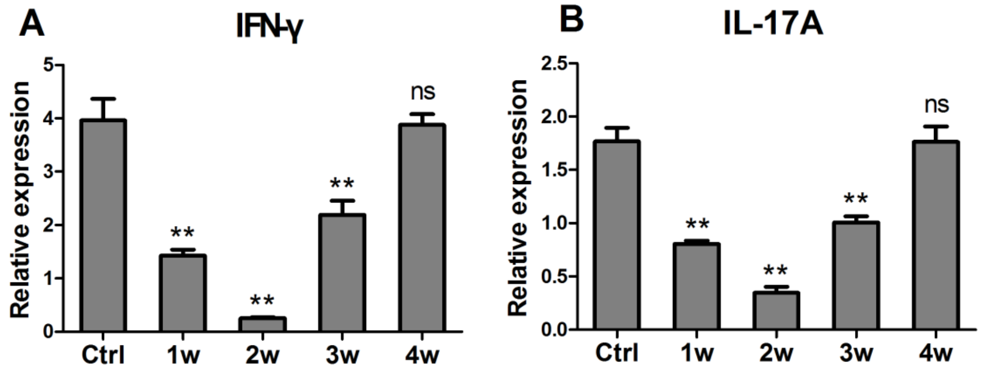

Supplementary Figure.1 The expression of IFN-γ and IL-17A mRNA in the spleen of EAM mice. (A) As compared with the controls (Ctrl), the relative expression of IFN-γ mRNA was decreased most in the spleen of EAM mice at 2 weeks (w) after the first immunization. (B) Compared with the controls, the relative expression of IL-17A mRNA in the muscle of EAM mice was significantly decreased most in the spleen of EAM mice at 2 weeks after the first immunization. $^{**}P < 0.001$, $^{ns}P > 0.05$,vs the controls (Ctrl).
