# Supplementary material for: Toll-Like Receptor 4–Myeloid Differentiation Primary Response Gene 88 Pathway Is Involved in the Inflammatory Development of Polymyositis by Mediating Interferon-γ and Interleukin-17A in Humans and Experimental Autoimmune Myositis Mouse Model
Source: Front Neurol. 2017 Apr 12;8:132. doi: 10.3389/fneur.2017.00132 (PMC5388689; doi:10.3389/fneur.2017.00132)
Supplement: Supplementary file 3 [file Image_2.PDF]

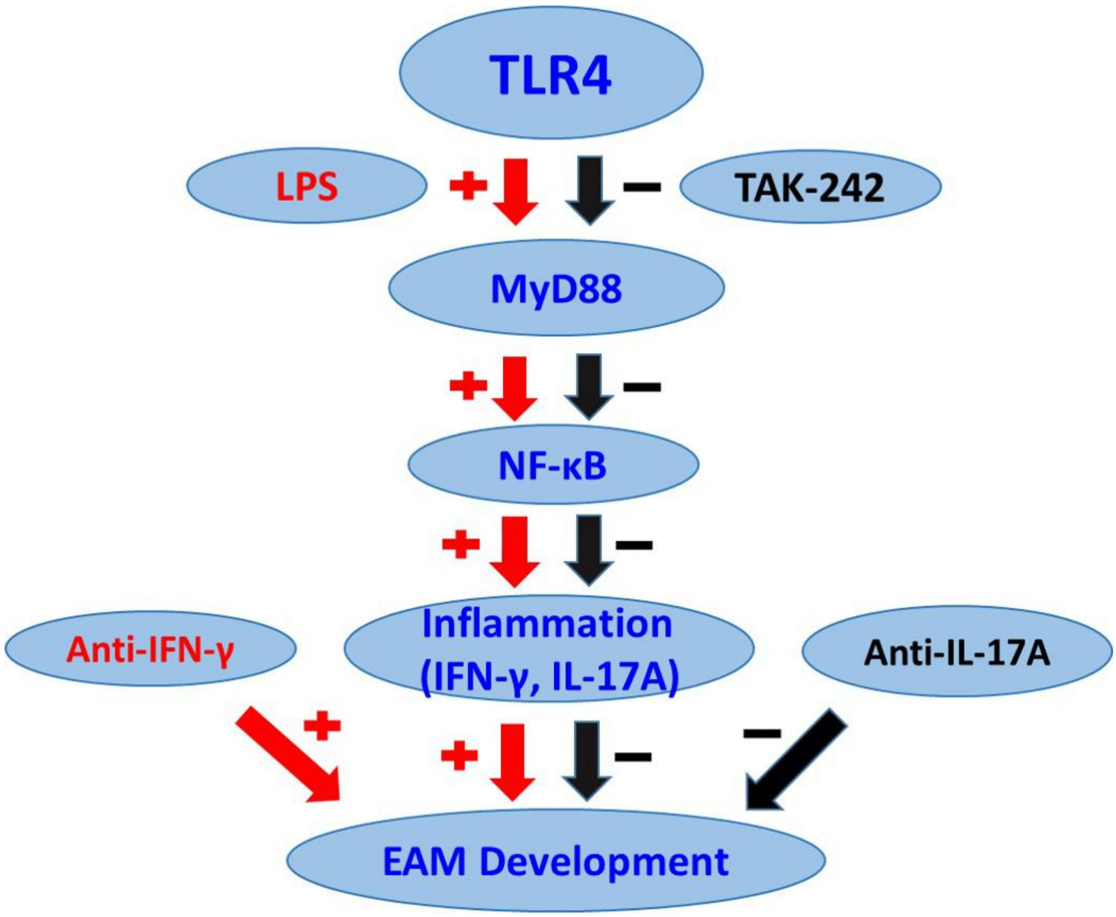

Supplementary Figure.2 TLR4-MyD88 pathway was involved in the development of EAM by mediating IFN-γ/IL-17A. TLR4 agonist LPS activated TLR4-MyD88 -NF-κB pathway, induced the production of inflammatory cytokines (such as IFN-γ and IL-17A) and exacerbated the development of EAM. But TLR4 antagonist TAK-242 inhibited TLR4-MyD88 pathway and mitigated the inflammatory development of EAM. The neutralization of anti-IFN-γ antibody exacerbated EAM inflammation and the neutralization of anti-IL-17A antibody mitigated EAM inflammation.
